# Recognizing the Emergent and Submerged Iceberg of the Celiac Disease: ITAMA Project—Global Strategy Protocol

**DOI:** 10.3390/pediatric14020037

**Published:** 2022-06-10

**Authors:** Giuseppe Magazzù, Samuel Aquilina, Christopher Barbara, Ramon Bondin, Ignazio Brusca, Jacqueline Bugeja, Mark Camilleri, Donato Cascio, Stefano Costa, Chiara Cuzzupè, Annalise Duca, Maria Fregapane, Vito Gentile, Angele Giuliano, Alessia Grifò, Anne-Marie Grima, Antonio Ieni, Giada Li Calzi, Fabiana Maisano, Giuseppinella Melita, Socrate Pallio, Ilenia Panasiti, Salvatore Pellegrino, Claudio Romano, Salvatore Sorce, Marco Elio Tabacchi, Vincenzo Taormina, Domenico Tegolo, Andrea Tortora, Cesare Valenti, Cecil Vella, Giuseppe Raso

**Affiliations:** 1 Dipartimento di Patologia Umana dell’Adulto e dell’Età Evolutiva “Gaetano Barresi”, Università di Messina, 98122 Messina, Italy; chiaracuzzupe@gmail.com (C.C.); piccolaalessia93@hotmail.it (A.G.); antonio.ieni@unime.it (A.I.); fabianamaisano@yahoo.it (F.M.); giuseppinella.melita@unime.it (G.M.); ileniapanasiti89@gmail.com (I.P.); claudio.romano@unime.it (C.R.); 2 Department of Paediatrics, Mater Dei Hospital, 2090 Msida, Malta; samuel.aquilina@gov.mt (S.A.); ramon.bondin@gmail.com (R.B.); grima.annemarie@gmail.com (A.-M.G.); cecil.vella@gov.mt (C.V.); 3 Department of Pathology, Mater Dei Hospital, 2090 Msida, Malta; christopher.barbara@gov.mt (C.B.); mark.g.camilleri@gov.mt (M.C.); 4 Ospedale Fatebenefratelli, Buccheri La Ferla, 90123 Palermo, Italy; ignbr@libero.it (I.B.); maria.fregapane@libero.it (M.F.); 5 AcrossLimits Ltd., 4013 Birkirkara, Malta; jacqueline@acrosslimits.com (J.B.); info@annaliseduca.me (A.D.); angele@acrosslimits.com (A.G.); 6 Dipartimento di Fisica e Chimica-“E. Segrè”, Università di Palermo, 90133 Palermo, Italy; donato.cascio@unipa.it (D.C.); vito.gentile@unipa.it (V.G.); giadalicalzi@gmail.com (G.L.C.); salvatore.sorce@unipa.it (S.S.); taormina.maltese@gmail.com (V.T.); giuseppe.raso@unipa.it (G.R.); 7 DAI Materno-Infantile, AOU Policlinico G. Martino, 98124 Messina, Italy; stefan.costa@gmail.com (S.C.); fumonero@inwind.it (S.P.); 8 Dipartimento di Medicina Clinica e Sperimentale, Università di Messina, 98122 Messina, Italy; socrate.pallio@unime.it; 9 Facoltà di Ingegneria e Architettura, Università degli Studi di Enna “Kore”, 94100 Enna, Italy; 10 Dipartimento di Matematica e Informatica, Università di Palermo, 90133 Palermo, Italy; metabacchi@gmail.com (M.E.T.); domenico.tegolo@unipa.it (D.T.); cesare.valenti@unipa.it (C.V.); 11 DAI Scienze Mediche, AOU Policlinico G. Martino, 98124 Messina, Italy; andrea.tortora@polime.it

**Keywords:** coeliac disease, anti-transglutaminase, mucosal deposits, point-of-care test, negative predictive value, artificial intelligence, intestinal biopsy, ESPGHAN, guidelines

## Abstract

Coeliac disease (CD) is frequently underdiagnosed with a consequent heavy burden in terms of morbidity and health care costs. Diagnosis of CD is based on the evaluation of symptoms and anti-transglutaminase antibodies IgA (TGA-IgA) levels, with values above a tenfold increase being the basis of the biopsy-free diagnostic approach suggested by present guidelines. This study showcased the largest screening project for CD carried out to date in school children (n=20,000) aimed at assessing the diagnostic accuracy of minimally invasive finger prick point-of-care tests (POCT) which, combined with conventional celiac serology and the aid of an artificial intelligence-based system, may eliminate the need for intestinal biopsy. Moreover, this study delves deeper into the “coeliac iceberg” in an attempt to identify people with disorders who may benefit from a gluten-free diet, even in the absence of gastrointestinal symptoms, abnormal serology and histology. This was achieved by looking for TGA-IgA mucosal deposits in duodenal biopsy. This large European multidisciplinary health project paves the way to an improved quality of life for patients by reducing the costs for diagnosis due to delayed findings of CD and to offer business opportunities in terms of diagnostic tools and support.

## 1. Introduction

Coeliac disease (CD) is an immune-mediated disorder against dietary gluten present in wheat, rye, and barley occurring in genetically susceptible individuals. It is also considered to be a systemic disorder characterized by a variable combination of gluten-sensitivity related signs and symptoms and disease-specific antibodies, which may ultimately result in enteropathy [1]. CD is frequently underdiagnosed and its consequent burden in terms of morbidity, mortality, and health care cost in the Mediterranean area has been reported [2]. The best available estimation of CD-associated medical cost was that carried out by Long et al. [3], reporting that the greatest annual medical costs in the years preceding the diagnosis of CD in comparison with those after the diagnosis are due to increased in-patient admissions, out-patient cost, laboratory tests, radiology, and office visits [3]. 

The diagnosis of CD relies on the clinical examination and suspicion raised by physicians followed by measurement of anti-transglutaminase antibodies IgA (TGA-IgA) and duodenal biopsy that shows compatible histologic damage [4]. Since 2012, the European Society for Paediatric Gastroenterology, Hepatology and Nutrition (ESPGHAN) has suggested that a tenfold increase in the level of TGA-IgA, together with further investigations and a strict protocol, are enough to diagnose coeliac disease without the need for duodenal biopsy [5]. In 2020, the no-biopsy approach for coeliac disease diagnosis was extended for children, even if asymptomatic, with TGA-IgA more than ten times the upper limit of normal (ULN) with the appropriate laboratory tests and positive Endomysial antibodies (EMA-IgA) from a repeated second serum sample. Children with positive TGA-IgA but lower titers (<10x ULN) should undergo biopsies to reduce the risk of false positive diagnosis [4]. In the pediatric age group, symptoms may not be reliable in the diagnosis of coeliac disease as described by Rosen et al. [6] and thus recommendations for reviewing additional CD screening criteria were suggested [7]. 

Recently, a study conducted by Gatti et al. highlighted an increased prevalence of CD in Italy by screening school children for HLA genes, associated with increased risk of celiac disease, and for total serum levels of IgA and IgA class anti-tissue transglutaminase in HLA-positive children [8]. More recently, the Autoimmunity Screening for Kids (ASK), a large scale pediatric screening study in Colorado for CD and type 1 diabetes, reported the CD outcomes for the first 9973 children screened through ASK [9]. Besides the high costs, especially if HLA gene testing is included, an important limiting factor in the pediatric population mass screening using conventional TGA-IgA may be the low compliance of asymptomatic children to be referred for usual serological testing. In 2007, Korponay-Szabo et al. [10] evaluated the feasibility and diagnostic accuracy of screening for coeliac disease by rapid IgA antibodies to tissue transglutaminase testing of finger-prick blood performed by district nurses in primary care [10]. Further advancements in Point-of-care tests (POCT) have been suggested as a possibility for a rapid and cheap tool for reducing the burden of coeliac disease in the Mediterranean area, especially in countries with limited resources [11]. Subsequently, a systematic review and meta-analysis concluded that the pooled sensitivity and specificity of POCTs in diagnosing CD are very high. These characteristics allow for the POCTs to be used as a screening tool for CD, especially in areas with limited access to laboratory-based testing [12]. Emphasis, however, must be made on the need for further studies to assess the right settings and the most convenient strategies for eliminating the underdiagnosis of CD and the need of invasive confirmatory procedures [12]. 

Presently, taking into account the recommendations on “who should be tested for CD” guidelines, the disease remains severely underdiagnosed [13]. A case study in the Netherlands Youth Health Care Centres, using a POCT to assess TGA-IgA, highlighted the fact that untreated CD has a considerable health burden on society [13]. Moreover, a preceding study had suggested that a mass screening using POCTs may be a useful and economic option for screening asymptomatic children and seems more convenient than a case finding strategy based on symptoms [14], contrary to what is observed in adults [15]. A literature review [12] showed that a critical point for using a POCT as screening tool is to define, with very narrow confidence limits, the negative predictive values of a test validated in a prospective way and to determine the reference standard to all subjects to whom a test has to be applied. 

Traditionally, CD, like other underdiagnosed disorders, is depicted like the proverbial tip of the iceberg [16], in which the largest and unknown part is submerged under water. Underdiagnoses of CD due to a low level of awareness and expertise needed to tackle the problem and the paucity of diagnostic resources only constitute to the visible tip of the iceberg. The full spectrum of the CD conundrum can only be completed once the hidden and unknown submerged part of the metaphorical “coeliac iceberg” is analyzed and understood completely. This is the most insidious and dangerous aspect because it includes subjects with most predominantly autoimmune diseases that are not considered related to CD because of the absence of gastrointestinal symptoms. Case in point are Dermatitis herpetiformis [17], Idiopathic Ataxia [18], Type-1 diabetes [19], and IgA Nephropathy [20]. All these disorders are generally characterized by the absence of gastrointestinal symptoms and negative coeliac serology and histology, but the presence of TGA-IgA deposits in the intestinal mucosa [17], in such cases, suggests the relationship with gluten ingestion. In two of these disorders, TGA-IgA deposits were also found in the target organs, brain and kidney, respectively [20,21].

Due to the complex nature of understanding CD, owing to a multitude of tests for diagnosis, varied clinical symptoms, significant underlying diseases, and inter-patient variability, the introduction of a Clinical Decision Support Systems (CDSS) may aid in improving diagnostic work-up, allowing for major cost, time, and work savings [22]. In particular, CDSSs based on fuzzy logic are nowadays a hot topic in research fields aimed at solving classification problems in a wide range of application areas, especially in medicine, where the possibility of presenting classification results together with a measurement of risk status is very achievable [23]. With the growing use of machine learning, the interest of the scientific community in the development of systems for the support of CD diagnosis is always on the rise [24]. The complexity of the diagnostic process of CD, however, has led to few research studies being conducted using CDSS vis-à-vis the diagnosis of CD. The ITAMA Project aimed to fill in this niche using a large target population in a small island in which all subjects at risk for CD can be easily reached. This scenario represents the ideal place to plan a mass screening study especially when the disease is largely underdiagnosed, hence Malta can be regarded as the epitome in mass screening testing grounds. 

The above mentioned critical points are developed by the ITAMA project, the characteristics of which are shown in Table 1. 

## 2. Aims and Hypothesis of the ITAMA Project

The specific aims of the study were:

1. To determine if a rapid and cheap POCT can bridge the diagnostic gap of CD in a large target population in Malta.
2. To evaluate the diagnostic accuracy of the POCT utilized in the study.
3. To analyze whether serial testing with POCT and conventional celiac serology may decrease the need for intestinal biopsy for diagnosing CD in children as still indicated by ESPGHAN.
4. To assess the negative predictive value of POCT.
5. To delve deep in the “coeliac iceberg” in order to identify people with biomarkers of potential gluten related disorders, such as TGA-IgA intestinal mucosal deposits (MD), who might benefit from a gluten-free diet.
6. To develop and validate an artificial intelligence-based system to support clinical decisions in CD diagnosis.
7. To investigate the potential cost savings resulting from the project.

Apart from being the largest CD screening program in school aged children carried out to date worldwide, the aims no. 3 to 6 in this study addressed and investigated CD diagnosis for the first time in the scientific literature. 

## 3. Methods

The project was developed in different settings and locations to address and answer all the study aims and hypothesis:

1. *Fulfilment of aims 1 to 3*—In Maltese primary schools and the general hospital, Mater Dei Hospital, all children with a suspicion of CD after a positive POCT result where referred for further secondary confirmatory tests.
2. *Fulfilment of aims 4 and 5*—In Sicily, at the Messina University Hospital Digestive Endoscopy Unit and the Regional Center for CD and at the Buccheri La Ferla Hospital in Palermo, centralized serology testing, both from Maltese and Sicilian patients, was performed.
3. *Fulfilment of aims 6 and 7*—Project coordination, leadership, and cost control, together with the development of the CDSS, were undertaken by the Physics and Chemistry Department “E. Segre” at the University of Palermo and AcrossLimits Ltd. in Malta respectively.

The individual methods for the three clinical settings are described in detail.

### 3.1. Study Protocol in Malta—School and Hospital Settings

#### 3.1.1. Target Population

Approval from the Directorate for Research, Lifelong Learning and Employability in Malta was granted to conduct the research in State Schools according to the official national rules and regulations, following the approval from the Ethics Committee of the respective Higher Educational Institution. 

The researchers were committed to comply with the General Data Protection Regulation (GDPR) and ensured that these requirements were followed in the conduct of this research. The researchers sent letters with clear information about the research, as well as consent forms to all data subjects and their parents/guardians when minors are involved. Further details about the policy for research in schools in Malta are provided at www.research.gov.mt (accessed on 1 January 2018).

Ethics approval was granted to screen up to 20,000 Maltese children between 3 and 13 years of age. An information leaflet was handed to the subjects through their respective schools. This also contained a questionnaire (Table 2) and consent form. Whoever chose to voluntarily participate would return the filled-up consent and tailored questionnaire back to the school. The testing phase started on the 11 March 2020.

#### 3.1.2. Initial Screening

A public tendering process was used to acquire coeliac POCT kits. The chosen POCT had a sensitivity of >99% and a specificity of 98.9% and was able to detect anti-tissue anti-transglutaminase antibodies (TGA) IgM, IgA, and IgG. Through another tender, forty nurses were recruited to perform the testing in schools and were instructed on how to perform the POCT and insert the anonymized information into a computer database by means of a user-friendly interface. The information retained included the answers of the questionnaire, the result of the POCT, as well as a photo of each POCT before and after the elapsed time of testing. The results were reviewed by a blinded panel of doctors who confirmed the results, and in case of a mismatch, a third judgement from a different doctor in the panel was entered. 

#### 3.1.3. Follow-Ups

All children who were found to have a positive POCT, as well as the children who had at least five symptoms from the questionnaire (Table 2), were called and offered further testing. Follow-up testing included serum total IgA, TGA-IgA levels, anti-Endomysial antibodies (EMA). TGA-IgA levels and EMA were determined at the Immunology Laboratory, Mater Dei Hospital utilizing certified IVD commercial kits (Eu-TTg IgA—ELISA EUROSPITAL^®^, Trieste, Italy—and IIFT Liver (monkey) IgA EUROIMMUN^®^, Lübeck, Germany). A duplicate serum was frozen and sent to the Buccheri La Ferla Hospital in Palermo for the retesting of same tests, with the addition of anti-Actin antibodies. In accordance with the ESPGHAN guidelines, children with TGA-IgA values higher than 10 times the ULN and positive EMA on a second serum sample taken on a separate occasion were diagnosed with CD, while those with positive TGA-IgA values but with lower than 10 times the ULN were offered endoscopy with duodenal biopsy and histological evaluation before CD diagnosis [5].

#### 3.1.4. Study Outcomes

The increase of CD prevalence. The number of intestinal biopsies that could have been avoided by serial testing. 

#### 3.1.5. Statistical Analysis

All laboratory tests, procedures, and validation of POCT were performed in light of the ESPGHAN guidelines and against the reference of EMA detected under standard conditions in an expert laboratory setting [5].

Diagnostic accuracy and post-test probability were calculated using the all-purpose 4-fold Table Analyzer and the interactive nomogram for post-test probability available on the Center for Evidence Based Medicine website (https://www.cebm.ox.ac.uk/resources/ebm-tools/catmaker-and-ebm-calculators (accessed on 1 February 2022)). The post-test probability was estimated for each test according to observed and expected prevalence in different settings, assuming as the pre-test probability of the second sample versus the post-test probability of the first one. The Odds ratios (OR) and 95% confidence intervals (CI) were evaluated. 

Taking into account ethical approval for 20,000 children to be screened and that 105 CD confirmed children were known in the eligible population of 51,845 children (prevalence 0.20%; 95% CI 0.17–0.24), we estimated to detect at least the same number of CD children by this screening. This, hypothetically, would lead to a significant increase in the new estimated prevalence, assuming a prevalence of CD in this population of 1%, a study power of 80%, a 92.3% POCT sensitivity, and a level of two-tail significance of 0.5%.

### 3.2. Study Protocol in Sicily—Digestive Endoscopy Unit Setting

#### 3.2.1. Study Participants

The protocol was approved by Ethics Committee of the University Hospital G. Martino, Messina. 

To assess the negative predictive value of POCT compared with that of coeliac conventional serology, patients at the Digestive Endoscopic Unit of the University Hospital in Messina who underwent endoscopic duodenal biopsy for reasons other than the suspicion of CD, such as dyspepsia, were enrolled. 

Following signed informed consent, patients were offered to fill in a questionnaire (Table 3) for symptoms and conditions associated with CD.

#### 3.2.2. Initial Testing and Procedures

POCT and blood drawing for conventional serology including TGA-IgA, anti-Actin antibodies, and EMA were conducted. 

The results of POCT, as well as a photo with the answers of the questionnaire, were anonymously inserted into the database. During the planned endoscopic exam, duodenal biopsy samples were taken for histology and with EMA quantification represented the reference standard. In subjects with symptoms or conditions associated with CD, a sample was frozen in optimal cutting temperature (OCT) and stored at −80 °C until use. The latter were then analyzed for TGA-IgA intestinal mucosal deposits (MD), the presence of which, regardless of serology and histology results, could suspect a relationship with gluten sensitivity [17]. 

#### 3.2.3. Confirmation Testing

TGA-IgA, EMA, and anti-Actin antibodies were centralized and blindly determined at Buccheri La Ferla Hospital in Palermo by commercial kits. Results and pictures of EMA were inserted into the database. Histology was blindly evaluated according to the modified Marsh classification [25].

Double immunofluorescence on duodenal mucosa was performed to detect mucosal deposits of anti-tissue transglutaminase type 2 (anti-tTG-2) using a slightly modified technique to that described by Karponay-Szabo et al. [17]. In summary, the technique involved obtaining 5 μm sections from the duodenal specimen embedded in the OCT compound and storing them at −80° in liquid nitrogen. Sections were fixed in acetone and incubated with Normal Rabbit Serum (Calbiochem, Darmstadt, Germany) for 20 min to block nonspecific sites. Sections were then incubated with anti-tTG-2 from mouse (CUB7402 from Neomarker, Fremont, CA, USA) for one hour, and then labelled with secondary antibodies conjugated with fluorochromes to detect total IgA (in green, using Polyclonal Rabbit anti-Human IgA/FICT from Dako, Glostrup, Denmark) and anti-tTG-2 (in red, using Polyconal Rabbit anti-Mouse RPE F (ab’) 2 from Dako, Denmark) for 30 min. The overlap of green and red fluorochromes produced a yellow fluorescence which indicated the deposits of anti-tTG2. Analysis was performed on confocal microscopy. 

#### 3.2.4. Study Outcomes

The Negative Predictive Value of POCT was evaluated against the gold standard performed in all subjects.

The prevalence of MD was assessed in subjects with clinical conditions associated with CD.

#### 3.2.5. Statistical Analysis

We expressed the results as NPV and as negative likelihood ratio (LR-). The Fagan nomogram (https://www.cebm.ox.ac.uk/resources/ebm-tools/catmaker-and-ebm-calculators (accessed on 1 February 2022)) was used to estimate LR and post-test probabilities. 

#### 3.2.6. Sample Size

Assuming a prevalence of CD in this population of 1.3% [26], a study power of 80%, a 92.3% POCT sensitivity, and a level of two-tail significance of 0.5, the minimum number of dyspeptic patients to enroll was 923 in order to obtain a NPV of 99.9%.

Initial testing planned in March 2020 had to be postponed due to the COVID-19 pandemic. The sample size calculation was re-evaluated and was later reduced to 500 subjects, lowering the NPV as a limitation due to the COVID-19 pandemic. 

### 3.3. Study Protocol in Sicily—Celiac Disease Center Setting

#### 3.3.1. Study Participants

The protocol was approved by Ethics Committee of the University Hospital G. Martino, Messina and started on 17 August 2020.

At the Regional Center for CD of the University Hospital in Messina, first degree relatives of confirmed CD patients at the Celiac Center were called up. Candidates were excluded if they underwent celiac serology determination in the last 2 years. Informed consent forms were given to patients who accepted to undergo upper digestive endoscopy, regardless the results of serology, and participate in the study.

#### 3.3.2. Initial Testing

POCT and blood drawing for conventional serology included TGA-IgA, anti-Actin antibodies, and EMA. 

The results of POCT as well as a photo, with the answers of the questionnaire, were anonymously inserted into the database. In case of positive POCT, or in case of negative POCT but in presence of symptoms or conditions associated with CD, patients were offered confirmatory testing through upper digestive tract endoscopy.

#### 3.3.3. Confirmatory Testing and Procedures

During the scheduled endoscopic exam, duodenal biopsy samples were taken for conventional histology and a sample was frozen in OCT compound and stored at −80 °C until testing for TGA-IgA intestinal mucosal deposits (MD). Histological results were issued according to Marsh classification and mucosal deposits were visualized with confocal microscopy described above. Results were compared with conventional CD serology, centralized and blindly performed at the Buccheri La Ferla Hospital Laboratory. 

#### 3.3.4. Study Outcome

Prevalence of CD in first-degree relatives of celiac patients. 

Quantifying potential of CD patients was on the basis that presence of MD acts as a forthcoming CD marker [27]. 

#### 3.3.5. Statistical Analysis

Using the Fisher’s exact test, the presence/absence of anti-tTG-2 MD demographic factors were statistically analyzed.

Regarding the first-degree relatives of celiac patients to enroll for estimating the Negative Predictive Value of POCT, we assumed a prevalence of CD in this population of 15% according to a previous study [28], a prevalence of 10% in the population of relatives we were going to investigate, a level of two-tail significance of 0.5, and the minimum number to enroll to have a study power of 80% was 363 subjects.

Limitations resulting from the C-19 pandemic meant that the study had to be postponed during the onset of the first cases in March 2020. During the following six months, accompanying relatives were not allowed to stay in the outpatient clinic and many elective surgeries and interventions were postponed in light of suboptimal clinical conditions. For this reason, a slow and backlogged restart in testing occurred in August 2020 up until March 2022. 

#### 3.3.6. Development and Validation of an AI-Based System to Support Clinical Decisions in CD Diagnosis

A CDSS based on a neural network fuzzy classifier for CD diagnosis is one of the major outputs of the ITAMA project. CDSS developed in ITAMA was trained and tested both on a set of specifically generated simulated data, as well as on the real-world data gathered within the project. This was possible by creating a large database with positive and negative CD patient information. This database consisted of a set of examples (patterns) in the form of numerical characteristics obtained from the heterogeneous data originating from the tests performed. This would allow the AI system in the training phase to recognize and identify the optimal separation scenarios between the positive and negative cases. Once this phase was completed, around 20% of the total cases excluded from the training phase were used for the CDSS AI validation, which included the quantification of CD risk associated with these cases. Once complete data were inserted, the CDSS could assess the patient’s associated risk status, expressing it using a five point, Likert-like scale, from “very low” to “fairly high”, derived from the fuzzy output of the classifier. Clinicians could then use such information to augment this information with that of a physical patient examination. 

#### 3.3.7. Analysis of the Costs Saved as Consequence of the Project

The ITAMA Project aims to provide evidence and information to support decision makers in reallocation of resources from the outpatient setting to the screening/primary care setting, considering the following factors:

○ Increased avoidance of conventional serology and invasive procedures, such as upper digestive endoscopy with intestinal biopsy and increment of diagnosis.
○ Better reduction of diagnostic timeframes.
○ Reduction of outpatient consultations.
○ Reduction of social costs, such as loss of work and school days (however this is compensated with the increase in voucher volume).

In terms of cost analysis, the ITAMA project aims to evaluate:

○ whether a screening action is feasible, cost-effective, and well accepted by the population,
○ both possible disinvestments and investments that improve outcomes,
○ costs for undiagnosed children in a case-finding action.

Costs of screening actions will be compared with the cost of diagnostics and treatment by standard care.

## 4. Results

Demographic data of 20,013 patients who underwent POCT are shown in Table 4. 

The whole distribution of children in the three age ranges for both sexes was significantly different at 95% CI: age range 03–06 years: 7893/20,013 = 0.394 (95% CI 0.387–0.401); age range 7–10 years: 8681/20,013 = 0.433 (95% CI 0.426–0.440); age range 11–14 years: 3439/20,013 = 0.171 (95% CI 0.166–0.177)

No significant difference was observed regarding ethnicity and CD familiality in the three age groups in both sexes.

Demographic data of 280 children according to EMA results are shown in Table 5. No statistical difference was observed between 122 children with EMA positive result and 158 with EMA negative results with respect to age distribution, ethnicity, and CD familiality.

**Figure 1:**
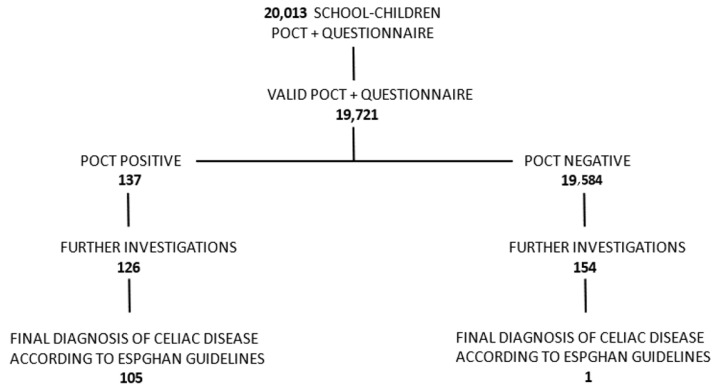
Diagram of study protocol in Malta, number of patients enrolled as of March 2022, and preliminary results.

The study began on the 11 March 2020. The number of subjects enrolled at this point and the numbers of subjects for each stage are shown in the flow chart for school-children in Malta, Figure 1. 

As of March 2022, a final diagnosis of CD was made, according to the ESPGHAN guidelines, in 106 children who underwent screening by POCT. Prior to the testing, a total of 105 known CD children were present in the eligible population, constituting a prevalence of 0.20% (95% CI, 0.17%–0.25%). This prevalence significantly increased to 0.42% (95% CI, 0.34%–0.52%) following this study.

**Figure 2:**
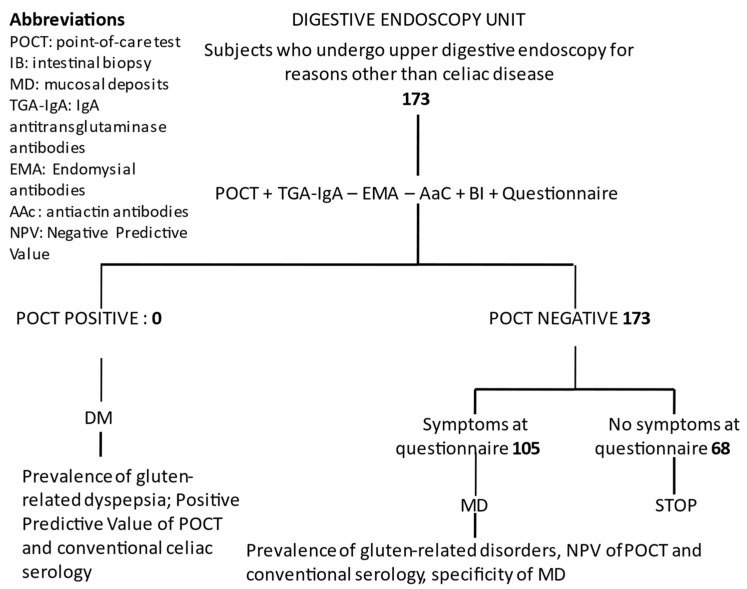
Diagram of study protocol in the Digestive Endoscopy Unit Setting, number of patients enrolled as of March 2022, and expected results.

**Figure 3:**
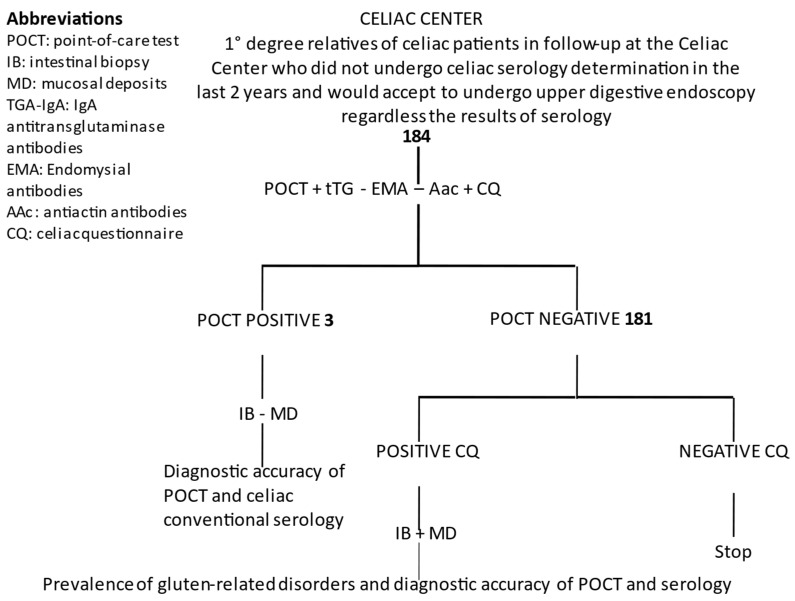
Study protocol in Sicily at the Celiac Center Setting, number of patients enrolled as of March 2022, and expected results.

With respect to the study protocol at the Digestive Endoscopy Unit and at the Celiac Center, the number of subjects enrolled at this point and the numbers of subjects for each stage are shown in Figure 2 and Figure 3, respectively. 

**Figure 4:**
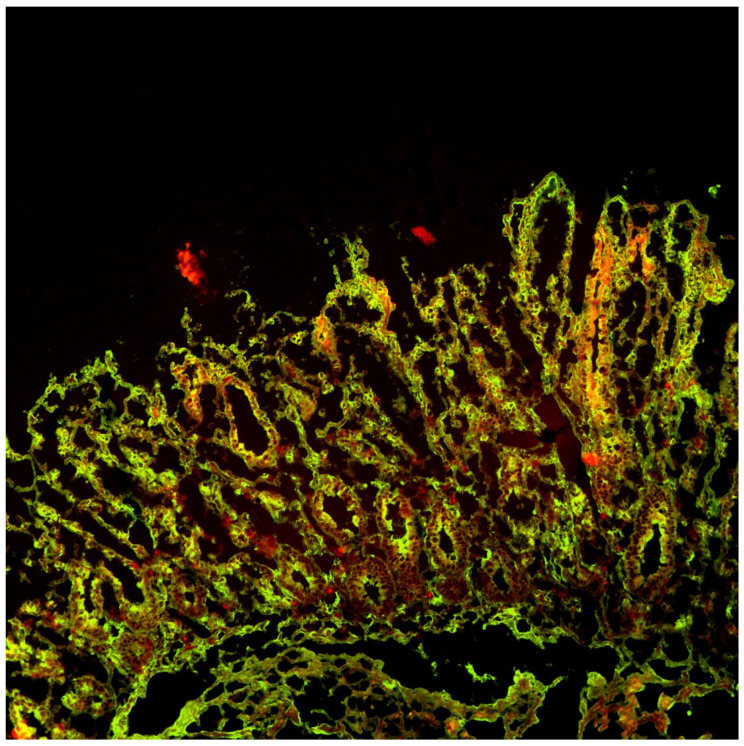
In yellow, the overlap of green and red indicates the deposits of anti-tTG2 on confocal microscopy.

**Figure 5:**
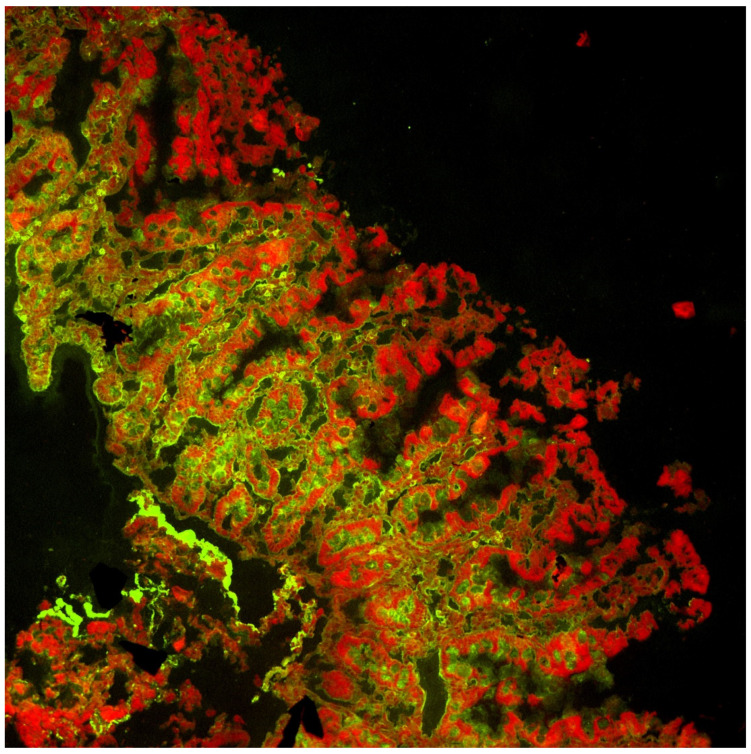
Deposition of IgA is shown in green, and deposition of TG2 is shown in red.

In Figure 4 and Figure 5 the presence of anti-tTG-2 MD are shown in yellow, whereas in their absence the red color of transglutaminase and the green color of IgA are observed individually. 

### Database Storing and CDSS Performances

The Database stores approximately 189,000 rows in total to date, including:

- 20,454 patient basic information, spread on 4 tables and 103,513 rows:
  ○ Demographic data (age, sex, ethnicity)
  ○ Medical history (answers to 29 multiple-choice questions)
  ○ Point-of-Care (POCT) data (pictures and results)
- 875 patient second-level exams results, spread on 12 tables and 2573 rows:
  ○ Blood tests (ant-TTG-IgA, anti-TTG-IgG, Total IgA, EMA(pictures), Anti-Actin AAC, anti-Deaminated Gliadin Peptide (DPG)-IgG)
- 165 patient third-level (endoscopy) exam results, spread on 2 tables and 228 rows:
  ○ Biopsy results (based on Marsh index)
  ○ Mucosal deposits (pictures and evaluation)
- 19,418 final diagnosis details, spread on 2 tables and 39,807 rows:
  ○ Diagnostic pathways (doctors’ decision on the diagnostic pathway for each participant)
  ○ Final diagnosis (Coeliac/Non-coeliac)

The database keeps track of all the collected data, including those not directly usable for the project’s goals but still useful for side statistics (defective POCTs or incomplete personal information). 

More importantly, 20,665 demographic details, responses to 20,556 questionnaires, and 20,519 POCT images are stored in the database. 

The amount of stored data is 95.8 GB, of which 31.4 MB corresponds to clinical and demographic data, and the remaining corresponds to indexed images of POCTs and MD. 

With its extensive training on real-world data contained in the database, the CDSS performances show a 99% accuracy, 86% sensitivity, 99% specificity, and 96% recall, values which compete with other state-of-the-art computational intelligence methods and systems. 

## 5. Discussion

The ITAMA project’s seven major aims were drafted to fill the gaps presently known in CD diagnosis. This was carried out by the introduction, validation, and extensive use of a POCT as a tool for bridging the diagnostic gap of CD that is worldwide largely underdiagnosed, with consequent short-term and long-term complications [2]. The results from the latter were coupled with an avant-garde CDSS system to define a strategy for making the diagnosis easier, less invasive, and feasible. Moreover, this project’s exploration of the hypothetical deep roots of the “coeliac iceberg” has been made possible, uncovering many disorders that may be prevented by recognizing a pathogenetic relationship with gluten [14].

The study identified an increased prevalence of CD in Malta when compared with historical data and highlights the underdiagnosis of such a disease. This increased prevalence of CD in the community is further supported by two recent studies excluding the presence of the DQ2/DQ8 haplotype HLA typing in non-CD patients [8,9]. Gatti et al. [8] showed an increased prevalence of CD in Italy when compared with results from past screening in schools. Stahl et al. [9] also presented an ongoing protocol for screening both CD and type-1 diabetes autoimmunity. Both the studies utilize conventional TGA-IgA antibodies as screening tool in subjects genetically compatible with CD. The current healthcare approach, despite the increasing prevalence and international guidelines for serological screening in appropriate patient cohorts [4,5], was unable to solve the underdiagnosis of CD. POCTs for CD detection, as for many other disorders, viruses, and diseases nowadays, have been developed over the past decade with the aim of improving case detection using rapid and convenient testing [10,13]. These POCT features allow for mass screening usage as highlighted by Korponay-Szabo et al. [10]. The study explored the feasibility of population screening for coeliac disease by means of a rapid antibody test performed by local healthcare workers in primary care setting. A different approach by Meijer-Boekel et al. [13] performed a case finding project to detect CD children who visit the Youth Health Care Centres in the Netherlands to evaluate whether it is feasible, cost-effective, and well accepted by the population [13]. A study compared these two different approaches and concluded that a mass screening through POCT seemed more convenient than a case finding strategy, based on symptoms, that otherwise can miss asymptomatic children [14]. For optimal results as a screening tool, the POCT must have a high negative-predictive value in order to avoid missing cases. Literature analysis on POCT detecting anti-DGP IgA/IgG antibodies performed well in a population with high prevalence of CD [28], but showed a low sensitivity [29] in prospective studies. For this reason, the selection of a suitable POCT was a long and laborious procedure. Out of the nine testing POCT kits, five were automatically excluded because the sensitivity and specificity was less than the requested limit; literature review showed that validation of the POCT was conducted on less than 500 subjects; the POCT was not mentioned in any literature studies; or did not detect IgA deficient patients. The final four kits were evaluated against 10 known CD patient and 10 non-CD patients using a finger-prick sampling method, as intended by all POCT instructions. One kit (BioHit Healthcare^®^ Celiac Quick test, Helsinki, Finland) showed 100% PPV and NPV to the tests subjects, including one borderline positive TTG-IgG patient, which was undetectable by the other three kits. The chosen POCT had a proven manufacturer sensitivity of >99% and a specificity of 98.9%, able to detect anti-TTG antibodies IgM, IgA, and IgG. Apart from the diagnostic accuracy claimed by the manufacturer, the NPV must be assessed in a prospective way and determining the reference standard to all subjects to whom a test must be applied. There is clearly a potential workup bias in studies where the gold standard test is performed only on people who have already tested positive for the test being validated [30]. The assessment of the POCT NPV in this study was performed in a population such as dyspeptic patients who all undergo the reference standard, which is duodenal histology. 

Large-scale POCT validation was conducted in a population which includes mild and severe disease, treated and untreated subjects, and those with different but commonly confused conditions. In this case, the larger the sample, the narrower the confidence interval [30]. The study conducted and validated the POCT in one of the largest screening projects for CD so far, comprising more than 20,000 children, which is 40% of the eligible population in Malta. 

The study also sought to answer whether the association of more tests may allow the avoidance of EMA analysis and intestinal biopsy even in cases where <10x TGA-IgA ULN is primarily seen. This aspect can make the diagnosis easier, cheaper, and feasible even in countries with limited resources.

With respect to the spectrum of disorders potentially related to gluten ingestion, searching for MD in a large cohort, apart from the already known disorders such as dermatitis herpetiformis, ataxia, type-1 diabetes, and IgA nephropathy [17,18,19,20], our study may identify other autoimmune disorders that, in presence of MD in the intestine, may benefit from a gluten-free diet.

The large dataset found in the CDSS database is regarded as one of the major outputs of the study. Apart from offering clinical solutions represented as some of the patient results showcased in this study, this archive of results can be used as a foundation to other similar studies in the field of CD diagnosis or relatable disorders. 

Finally, the cost analysis, taking into consideration both health and social outcomes, deriving from the adoption of a POCT, may suggest switching the diagnostic pathway for CD from a hospital outpatient setting to the pediatrician’s office.

### Limitations

POCT may have different diagnostic accuracy in children and adults, the latter possibly having a different TGA-IgA response to gluten. Assessing its negative predictive value in a population of adults could not correspond to that of children. This issue was tackled by performing conventional serology also in children with negative POCT with more symptoms utilizing EMA, the gold standard, according to the ESPGHAN guidelines. A small fraction of symptomatic children accepted to undergo further investigations after POCT resulted negative, probably being reassured by the initial result. 

Preliminary results showed a doubled prevalence of CD in Malta through this screening but figures are still lower (0.42%) than those expected according to other mass screenings performed in other Caucasian populations [31]. This result is not a limitation of the POCT utilized, as field testing results showed a 99% NPV. A future recommendation in a different age group cohort population including the rest (60%) of the eligible population could permit another significant bridge in the diagnostic gap for CD in Malta.

The COVID-19 pandemic has also brought about restrictions in the target population size, especially in the Sicilian cohort. Restrictions in clinic use and the number of elective operations conducted has meant that the original population sizes could not be honored. The target population in Malta was achieved by a substantial prolongation of the testing timeframes and the addition of more human resources and facilities to complete preset targets. 

## 6. Conclusions

Health can, and must, be considered as one of the strategic themes for projects dealing with territorial and cross-border development. 

Multidisciplinary healthcare projects are strategic in three aspects:

- They improve the quality of life of the inhabitants of the territory by often facing common challenges in cross-border regions.
- Where possible, they reduce the costs for diagnosis and those due to delayed diagnosis.
- Furthermore, they offer job opportunities to companies in the area that deal with diagnostic tools or diagnostic support.

Although several important topics have arisen from this study, the preliminary results suggest that an extension of the project, both in terms of age of subjects to enroll and duration, may be needed to bridge the diagnostic gap of CD and to reduce the burden of the disease. 

## Figures and Tables

**Table 1:** The ITAMA project.

The ICT (Information and Communication Technologies) Tools for the diagnosis of Autoimmune diseases (AD) in the Mediterranean Area (ITAMA) is an INTERREG V-A Italia—Malta Cooperation Project funded by the European Regional Development Fund. The Program Investment Priority axis is to “Promote the sustainable and smart growth through research and innovation” with specific objective to “Increase the innovation and research activities to improve the quality of life and cultural heritage fruition”.
The **Common Challenge** is enhancing health and quality of life by improving the diagnosis of AD, third in the world after cardiovascular and cancer in terms of incidence, with a focus on the study of celiac disease in the Mediterranean.
The **Overall Objective** is to activate a network between research and productive environments in the healthcare sectors to develop innovative ICT Tools for the diagnosis of AD, and related technology transfer tools. Expected change concerns the anticipation of diagnosis time through the optimization of the diagnostic path.
The two **main outputs** produced are: Database and innovative ICT tools to support the diagnosis of celiac disease for Healthcare delivery services; Technology transfer services by modeling production processes based on the project’s results for health companies and specialized enterprises.
The **adopted approach** is: - **interdisciplinary** (doctors, biologists, physicists, computer scientists, engineers) - **cooperative** (universities, hospitals, institutions, PMI) - **cross-border**: the AD have both genetic and environmental risk factors; the advantage of comparative analysis in places with similar populations but in those with different lifestyles, such as in Sicily and Malta, improves understanding of their pathogenesis in relation to genetic and environmental profiles.
Project is **innovative** in three aspects: - **structural**: currently, data interpretation of some diagnostic tests is subjective and requires a double reading. Databases with heterogeneous data obtained from the tests performed for the diagnosis of AD and in particular celiac disease may be made available to the scientific community for epidemiological studies, development of automated diagnostic systems, and knowledge transfer. - **procedural**: validation of tools developed in the project. - **technological**: validation of an artificial intelligence-based system to support clinical decisions in celiac disease’s diagnosis has to be developed.

**Table 2:** Questionnaire utilized in Malta.

Do You Have Any Family Relatives with Coeliac Disease?			
Yes|No|Unknown|Father|Mother|Paternal Grandfather|Paternal Grandmother			
Maternal Grandfather|Maternal Grandmother|Sister|Brother|Other:			
1. Persistently tired/weak/low energy	Yes	No	Unknown
2. Immunodeficiency	Yes	No	Unknown
3. Vomiting (more than 1 episode per month in last 3 months)	Yes	No	Unknown
4. Liver problems	Yes	No	Unknown
5. Diabetes (type 1)	Yes	No	Unknown
6. Anaemia (pallor, low blood level)	Yes	No	Unknown
7. Rheumatoid Arthritis	Yes	No	Unknown
8. Renal problems	Yes	No	Unknown
9. Epilepsy	Yes	No	Unknown
10. Severe dental decay	Yes	No	Unknown
11. Mood changes	Yes	No	Unknown
12. Persistent loose stools	Yes	No	Unknown
13. Repeatedly complains of abdominal pain	Yes	No	Unknown
14. Thyroid problems	Yes	No	Unknown
15. Abdominal distention/bloating, flatulence	Yes	No	Unknown
16. Irregular bowel habits	Yes	No	Unknown
17. Alopecia (hair loss)	Yes	No	Unknown
18. Vitiligo (white skin patches)	Yes	No	Unknown
19. Down’s, Williams or Turner’s syndrome	Yes	No	Unknown
20. Recurrent Mouth Ulcers	Yes	No	Unknown
21. Difficulty with balance/walking	Yes	No	Unknown
22. Poor weight gain, anorexia, weight loss	Yes	No	Unknown
23. Short stature/growth failure	Yes	No	Unknown
24. Weak bones	Yes	No	Unknown
25. Constipation	Yes	No	Unknown

**Table 3:** Questionnaire utilized in Sicily.

1. Weakness/fatigue	Yes	No	Unknown
2. Total IgA deficiency	Yes	No	Unknown
3. Isolated and persistent hyper-transaminasemia (ALT-AST level two times the normal range for at least 3 months)	Yes	No	Unknown
4. Insulin-dependent type I diabetes	Yes	No	Unknown
5. Anaemia	Yes	No	Unknown
6. Rheumatoid Arthritis	Yes	No	Unknown
7. IgA nephropathy	Yes	No	Unknown
8. Epilepsies resistant to pharmacological treatment or epilepsies with intracranic calcification	Yes	No	Unknown
9. Teeth enamel defects	Yes	No	Unknown
10. Depression (treated with drugs)	Yes	No	Unknown
11. Chronic diarrhoea and/or malabsorption	Yes	No	Unknown
12. Repeatedly complains of abdominal pain (IBS)	Yes	No	Unknown
13. Thyroid disorders with positive antibodies	Yes	No	Unknown
14. Abdominal distention/bloating, flatulence (IBS)	Yes	No	Unknown
15. Irregular bowel habits (IBS)	Yes	No	Unknown
16. Alopecia	Yes	No	Unknown
17. Vitiligo	Yes	No	Unknown
18. Down syndrome and Turner syndrome	Yes	No	Unknown
19. Recurrent aphtous stomatitis (more than four episodes/year)	Yes	No	Unknown
20. Ataxia	Yes	No	Unknown
21. Weight loss	Yes	No	Unknown
22. Short stature	Yes	No	Unknown
23. Osteopenia (Z score < 2 S.D.)	Yes	No	Unknown
24. Constipation	Yes	No	Unknown
25. Chronic or recurrent joint pain (at least six times/year)	Yes	No	Unknown
26. Non-Hodgkin intestinal lymphoma	Yes	No	Unknown
27. Infertility and/or multiple miscarriage	Yes	No	Unknown
28. Other autoimmune disorders (such as systemic erythematosus lupus, etc.) with confirmed diagnosis at II or III level regional hospital	Yes	No	Unknown
29. Dermatitis herpetiformis (even if only suspected)	Yes	No	Unknown

**Table 4:** Demographic data of 20,013 children who underwent POCT in Malta.

SEX No.	FEMALES 10,025			MALES 9988		
AGE RANGE (Years)	3–6	7–10	11–14	3–6	7–10	11–14
No.	3921	4304	1800	3972	4377	1639
%	39.1	43.3	17.1	39.7	43.8	16.4
ETHNICITY						
CAUCASIAN/OTHER	3648/273	3990/314	1676/124	3700/272	4070/307	1512/127
% of Caucasian	93	92.7	93.1	93.1	92.9	92.2
CD FAMILIALITY						
YES/NO	421/3500	423/3881	213/1587	400/3572	398/3979	160/1479
%	10.7	9.6	11.8	10.1	9.1	9.7

**Table 5:** Demographic data according to endomysial (EMA) results of 280 children who underwent further investigations in Malta.

EMA No.	POSITIVE 122						NEGATIVE 158					
SEX No.	FEMALES 77			MALES 45			FEMALES 91			MALES 67		
AGE RANGE (Years)	3–6	7–10	11–14	3–6	7–10	11–14	3–6	7–10	11–14	3–6	7–10	11–14
No.	33	33	11	19	17	9	25	37	29	14	37	16
ETHNICITY												
CAUCASIAN/OTHER	31/1	33/0	10/1	18/1	15/2	9/0	25/0	33/4	29/0	13/1	32/5	14/2
CD FAMILIALITY												
YES/NO	7/25	4/29	2/9	4/15	4/13	1/8	3/22	7/30	7/22	4/10	3/34	4/12
